# Impact of allyl-isothiocyanate and high sucrose diet on antimicrobial peptide expression and survival in *Drosophila melanogaster*

**DOI:** 10.3389/fimmu.2026.1804605

**Published:** 2026-04-10

**Authors:** Sonja Dähn, Christian Zimmermann, Ronja Merschmann, Paul Schmutzler, Anika E. Wagner

**Affiliations:** 1 Institute of Nutritional Science, Justus Liebig University, Giessen, Germany; 2 Laboratory for Sensory Analysis and Consumer Research, Faculty of Agricultural Sciences, Georg August University, Göttingen, Germany; 3 Center for Sustainable Food Systems, Justus Liebig University, Giessen, Germany

**Keywords:** AITC - allyl-isothiocyanate, antimicrobial peptides (AMP), bioactive plant compound, *Drosophila melanogaster*, gene expression, high sucrose diet (HSD), infection, innate immunity

## Abstract

The global rise of antibiotic-resistant bacteria highlights the urgent need for alternative strategies to support host defense against infections. Bioactive plant-derived compounds, such as isothiocyanates, have gained attention due to their antimicrobial and immunomodulatory properties. Allyl-isothiocyanate (AITC), a hydrolysis product of glucosinolates found in Brassica vegetables, has demonstrated antimicrobial activity *in vitro* and the ability to modulate antimicrobial peptide (AMP) expression in cell culture models. However, its *in vivo* effects under metabolically challenging dietary conditions remain poorly understood. In this study, we investigated the impact of dietary AITC supplementation on immune responses and survival in *Drosophila melanogaster* exposed to a high-sucrose diet (HSD), a dietary condition known to impair metabolic health and immune function. Flies were fed a HSD with or without 0.25 mM AITC and subsequently subjected to oral infection with either *Leuconostoc pseudomesenteroides* or *Pectobacterium carotovorum* subsp. *carotovorum*, which preferentially activate the Toll and Imd signaling pathways, respectively. AMP expression was analyzed by qPCR and RNA sequencing, and physiological consequences were assessed by performing lifespan analysis. AITC did not affect the flies’ feed intake or basal AMP expression under non-infected conditions. HSD significantly shortened the lifespan in both sexes, and AITC supplementation was not able to rescue this effect. Following oral infection of the flies, both HSD alone and HSD supplemented with AITC influenced the survival in a sex- and pathogen-specific manner, with AITC frequently exacerbating mortality rather than improving outcomes. While selected AMPs, particularly *Attacin D*, were modulated in a context-dependent manner, RNA sequencing revealed no consistent transcriptional changes in core Toll or Imd pathway components. Overall, our findings indicate that dietary AITC does not enhance host defense in *Drosophila melanogaster* under high-sugar conditions and that its effects on immunity and survival are strongly sex-specific. These results highlight the complexity of diet–immune interactions and caution against extrapolating *in vitro* antimicrobial effects to *in vivo* host protection.

## 1 Introduction

The extensive use of antibiotics in both human and veterinary medicine has substantially contributed to the rise of antibiotic-resistant pathogenic bacteria (1). In the *Global Antimicrobial Resistance Surveillance Report* published by the WHO in 2025, it is reported that in 2021, 1.14 million deaths were associated with antibiotic resistance (2, 3). Consequently, developing novel therapeutic approaches and preventive strategies has become increasingly critical in addressing severe bacterial diseases. In this context, bioactive plant-derived compounds are gaining attention as promising alternatives in the management of bacterial infections. This includes, among others, glucosinolates, a group of bioactive plant compounds that are mainly present in plants of the genus *Brassica*, such as broccoli, cauliflower, radish and mustard (4). Glucosinolates are enzymatically hydrolyzed after tissue disruption induced by e.g. cutting, resulting besides others in the formation of isothiocyanates (ITCs) (5). One well investigated ITC is allyl-isothiocyanate (AITC), which is described to exhibit antioxidative, anticancer, anti-diabetic as well as antimicrobial activities (6–8). AITC has been reported to inhibit the growth of different pathogenic bacterial strains *in vitro*, including *Campylobacter jejuni* (9), *Listeria monocytogenes* and *Escherichia coli* (7). In intestinal epithelial cells ITCs have been shown to increase the mRNA expression of antimicrobial peptides (AMPs) such as human β-defensin-2 (10). AMPs are an essential component of the innate immune defense and are synthesized by a wide range of organisms, including mammals, insects, and plants (11). These predominantly cationic molecules exert their antimicrobial activity through both membrane-disruptive and non–membrane-targeting mechanisms, enabling them to eliminate bacteria, fungi, and viruses. In recent years, isolated AMPs have increasingly been considered as promising candidates for alternative therapeutic approaches in the treatment of bacterial infections (1).

Besides antibiotic resistance, obesity, promoted by overeating and sedentary lifestyle, represents an increasing challenge for human health. Obesity does not only cause metabolic disorders but also induce low-grade inflammation affecting multiple aspects of immune function. It has been shown that diet induced obesity leads to a dysregulated AMP production (12, 13) while a Western-style diet, frequently involved in the development of obesity, may induce a dysfunction of AMP-secreting Paneth cells in the intestine (14). As AITC is not only known to have antimicrobial effects, but also anti-diabetic properties, we hypothesize that AITC can ameliorate a high sugar diet-induced immunity disruption resulting from a modulation of AMP expression. To address this hypothesis, we used *Drosophila melanogaster* as model organism which has initially been mainly utilized for genetic research. Over the last decade, *D. melanogaster* has also been established as a valuable research model to address physiological research questions including for example food-induced disorders, microbiota and immunity (15). Due to its cost-effective and easy handling, short generation time and the fact that about 70% of their genes have orthologs in humans, it is an ideal model to investigate interactions between bioactive food compounds and the immune system (16). Similar to humans, where AMP expression is controlled by toll-like-receptors, AMP expression in *D. melanogaster* is controlled by two NFκB pathways, Toll and Imd (17–19).

To examine the impact of oral AITC treatment on the Toll- and Imd-pathway under high-caloric conditions, *D. melanogaster* were fed a high sucrose diet (HSD) containing 0.25 mM AITC. In addition, flies were also exposed to fly pathogenic bacteria, in particular *Pectobacterium carotovorum* subsp. *carotovorum* (ECC), known to induce Imd-signaling pathway, and *Leuconostoc pseudomesenteroides* (LP), known to activate the Toll-signaling pathway (20, 21). AMP expression levels were analyzed by mRNA sequencing under basal conditions and by qPCR under both basal and infected conditions. AMPs were selected on their postulated regulation by Toll and Imd signaling. *Drs* (*Drosomycin*) was analyzed as a Toll pathway target, whereas *AttD* (*Attacin-D*) and *DptA* (*Diptericin A*) represent Imd pathway dependent AMPs. *Mtk* (*Metchnikowin*) was included because it can be regulated by both Toll and Imd signaling, allowing assessment of potential pathway cross-talk. In addition, fly’s lifespan was determined as a physiological readout.

## 2 Material and methods

### 2.1 Husbandry of w^1118^ *Drosophila melanogaster*

Female and male w^1118^ *D. melanogaster* (Bloomington Drosophila Stock Center, Indiana, USA; #5905) were reared at 25 °C and 60% relative humidity, with a 12 h:12 h light–dark cycle in a climate-controlled chamber (Memmert HPP400, Büchenbach, Germany), on Caltech Medium (CT) (22). For all experiments, 3-day-old, age-matched adults originating from synchronized eggs were immobilized on ice and subsequently separated by sex and randomly allocated to the different diet groups. Groups of 25 flies each were transferred into vials (Ø 25 mm) containing either control medium (SY), high sucrose diet (HSD) or HSD containing 0.25 mM AITC. SY10 consisted of 10% sucrose (Carl Roth, Karlsruhe, Germany), 10% inactive yeast (Genesee via Kisker, Steinfurt, Germany), 2% agar (Apex via Kisker), as well as 0.3% propionic acid (Carl Roth) and 1.5% tegosept (Apex via Kisker) as preservatives. HSD consisted of 30% sucrose, instead of 10%. A 1-M stock solution of AITC (Sigma-Aldrich, Taufkirchen, Germany) was prepared in absolute ethanol (Merck, Darmstadt, Germany). To assess the effects of AITC, fly feed was supplemented with AITC at a final concentration of 0.25 mM, while an equivalent volume of absolute ethanol was added to the control medium as solvent control.

### 2.2 Gustatory assay

To evaluate potential effects of AITC on the feed intake of *D. melanogaster* a gustatory assay was performed as described previously (23). In brief, flies were maintained on either a control diet (SY), HSD or HSD supplemented with 0.25 mM AITC for 10 days. On day 10, flies were transferred to control medium, HSD or HSD with AITC (0.25 mM) stained with 0.2% sulforhodamine B sodium salt (Sigma-Aldrich) and kept under standard conditions for additional 8 hours. Subsequently, 20 flies per treatment group were collected in 200 µl PBS (pH 7.4; Thermo Fisher Scientific, Schwerte, Germany) containing 1% Triton X-100 (Sigma-Aldrich) and subsequently homogenized using a TissueLyser II (Qiagen, Hilden, Germany) at a frequency of 25 s^-^¹ for 6 minutes. The resulting homogenates were centrifuged (4,000 g, 5 min), and the fluorescence signal of the supernatant (excitation: 535 nm/emission: 590 nm) was measured using a SpectraMax iD3 microplate reader (Molecular Devices, San Jose, USA). Flies fed unstained feed served as background controls, and respective values were subtracted from the sample readings. A standard curve was generated using serial dilutions of sulforhodamine B sodium salt.

### 2.3 Bacterial cultivation and oral infection

**Figure 1:**
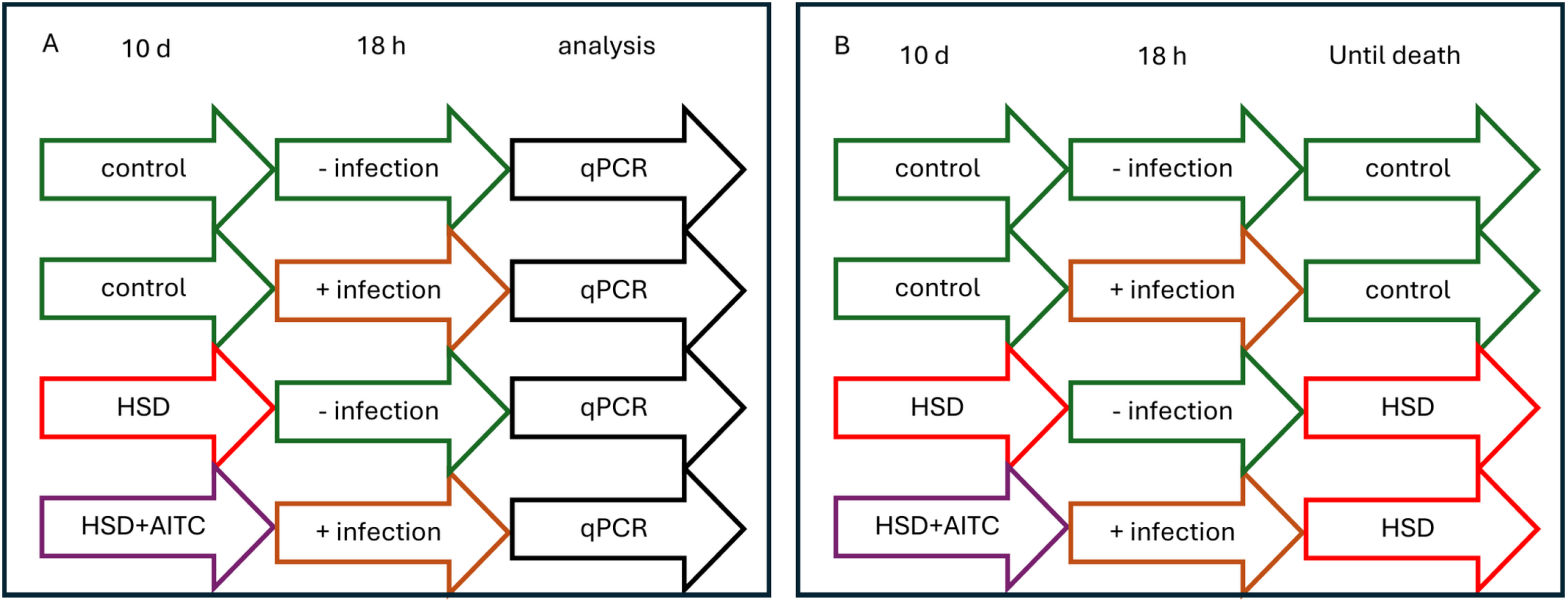
Experimental procedure of fly treatment for subsequent qPCR **(A)** and survival **(B)**. *D. melanogaster* were fed either control diet (SY) or high sucrose diet (HSD), with or without 0.25 mM allyl-isothiocyanate (AITC), for 10 days and treated either with sucrose solution or infected with *L. pseudomesenteroides* or *P. carotovorum* subsp. *carotovorum* for 18 h. For survival assays **(B)** the flies were transferred to the respective diet without AITC until the end of their lifes.

*Pectobacterium carotovorum* subsp. *carotovorum* (ECC) (Leibniz Institute DSMZ – German Collection of Microorganisms and Cell Cultures, Braunschweig, Germany) were grown in LB-Broth (Carl Roth), *Leuconostoc pseudomesenteroides* (LP) (a kind gift from Dr. Kwang-Zin Lee, Fraunhofer Institute for Molecular Biology and Applied Ecology, IME, Giessen, Germany) were grown in MRS-Broth (Carl Roth). Both bacterial strains were cultivated in a shaking incubator (B. Braun Biotech International, Melsungen, Germany) under aerobic conditions at 29 °C overnight. The following day, fresh medium was inoculated with the overnight bacterial culture and bacteria were grown under the same experimental conditions until they reached the stationary growth phase. For oral infection experiments, overnight cultures of ECC and LP were adjusted to an OD of 1 (corresponds to approximately 4.9 x 10^8^ CFU/ml for LP and 2.5 x 10^8^ CFU/ml for ECC (24, 25)) using a filter-sterilized (0.22 µm pore size, LLG-Syringe filter, Carl Roth) 100 mM sucrose solution. Subsequently, 1 mL of the bacteria–sucrose suspension or filter-sterilized sucrose solution (control) was applied to three layers of cellulose paper lining the bottom of each vial. Following a 10 days feeding on HSD and HSD supplemented with AITC, male and female *D. melanogaster* were transferred into the infection vials (25 flies per vial) and maintained under standard conditions for 18 h. After infection, the flies were either frozen at -80 °C for qPCR analysis (see **Figure 1A**) or transferred to fresh vials with the corresponding feed until the end of their life (see **Figure 1B**).

### 2.4 Survival analysis

To analyze the impact of HSD, AITC and bacterial infection on the flies´ survival, flies were treated as depicted in **Figure 1B**. After infection, flies were transferred to vials containing control feed only until the end of their life. Every second to third day, the animals were transferred to fresh vials and death events were recorded. Survival assays were repeated in 5 independent experiments. Each experiment consisted of 3 vials per treatment group, containing 25 flies each.

### 2.5 RNA-isolation

RNA was isolated by applying the Quick-RNA Tissue/Insect kit (Zymo Research, Freiburg, Germany), following the manufacturer’s instructions. In brief, 10 flies were homogenized in RNA lysis buffer. Following, the homogenate was transferred into a column tube subjected to several washing steps and treated with DNase. Finally, RNA was eluted in DNase/RNase-free water and stored at −80 °C until further use. The purity of the RNA samples was assessed photometrically at 260/280 nm using a UVmini-1240 UV–VIS spectrophotometer (Shimadzu, Duisburg, Germany). Samples with a 260/280 nm ratio below 1.7 were excluded from subsequent analyses.

### 2.6 qPCR

For a 2step qPCR, cDNA was initially synthesized from RNA samples. In detail, 1 µg of RNA was diluted in DNase-/RNase-free water to a final volume of 11.5 µl. 1 µl oligo(dT) primers (20 ng/µl) (Promega, Mannheim, Germany), 4 µl M-MLV RT 5× buffer (Promega), 0.5 µl RNasin RiboLock (40 U/µl) (Sigma-Aldrich), 2 µl dNTP mix (10 mM) (Promega), and 1 µl M-MLV reverse transcriptase (200 U/µl) (Promega) were added to the reaction mixture. Samples were incubated at 42 °C for 60 min, followed by heat inactivation in a thermocycler (T-Gradient ThermoBlock, Biometra, Göttingen, Germany) at 70 °C for 10 min. The resulting cDNA was stored at −80 °C until further use. qPCR master mix contained 10 µl PerfeCTa SYBR Green SuperMix, Low ROX (Quantabio, Beverly, MA, USA), 0.25 µl forward/reverse primer (10 pmol/µl), 7.5 µl DNase/RNase-free-water, and 2 µl cDNA. The qPCR was performed in a 7500 Real-Time PCR system (Applied Biosystems, Heidelberg, Germany). *RpL32* (*Ribosomal protein L32*, gene ID: 43573) and *RpS20 (Ribosomal protein S20, gene ID:* 42464) were used as reference genes. *Drs* (*Drosomycin, gene ID:* 38419), *Mtk* (*Metchnikowin*, gene ID: 36708), *AttD* (*Attacin-D, gene ID:* 42122) and *DptA* (*Diptericin A, gene ID:* 37183) were measured as target genes (for primer sequences refer to **Table 1**). The PCR data analyses were based on the 2^-ΔΔCT method described by (26). The geometric mean of the Ct values of the two housekeeping genes (*RpL32* and *RpS20*) was calculated and used for normalization. Flies were treated according to the procedure presented in **Figure 1A**.

**Table 1:** Primer sequences and annealing temperature for real-time PCR in *Drosophila melanogaster*.

Gene	Gene ID	Forward primer (5’ ➔ 3’)	Reverse primer (3’ ➔ 5’)	Temp.
*RpS20*	43573	TGTGGTGAGGGTTCCAAGAC	GACGATCTCAGAGGGCGAGT	58 °C
*RpL32*	42464	GGCAAGCTTCAAGATGACCA	GTTCGATCCGTAACCGATGT	55 °C
*AttD*	42122	CGGAGTAAGGGTCGGTGATG	GTGCATGACCATTGGCGTTG	58 °C
*Drs*	38419	TGCCTGTCCGGAAGATACAA	ATCCTTCGCACCAGCACTT	57 °C
*DptA*	37183	GAGATGCAGTTCACCATTGC	CCCTGAAGATTGAGTGGGTA	55 °C
*Mtk*	36708	CCTCATCGTCACCAGGGACC	TTGGACCCGGTCTTGGTTGG	55 °C

*RpS20*, Ribosomal protein S20; *RpL32*, Ribosomal protein L32; *AttD*, Attacin-D; *Drs*, Drosomycin; *DptA*, Diptericin A; *Mtk*, Metchnikowin.

### 2.7 RNA sequencing

RNA sequencing was conducted as an exploratory analysis to identify candidate genes responsive to dietary interventions under basal conditions. Based on these results, selected antimicrobial peptides were further examined by targeted qPCR analyses, including both basal and infection conditions. RNA was isolated from flies from all different treatments from three independent experiments, containing 10 flies each. The preparation of the RNA sample library, the sequencing of the RNA, as well as data collection were performed by Novogene Co., Ltd (Beijing, China). The quantity, quality, and integrity of the RNA were determined using an Agilent 5400 Fragment Analyzer System (Agilent Technologies, Santa Clara, USA). The mRNA was isolated from the total RNA by poly(A) capture. After library preparation, a 150 bp paired-end sequencing strategy was applied for sequencing. For this purpose, an Illumina NovaSeq X Plus PE150 (Illumina Inc., San Diego, USA) with a read depth of 9Gb/sample was used.

### 2.8 Statistics and illustrations

The statistical analysis of the RNA sequencing data was performed by Novogene Co., Ltd (Beijing, China). DESeq2 analysis followed by Wald test, identified significantly different expressed genes. The p-values were adjusted using the Benjamini-Hochberg method. Only genes with an adjusted p-value < 0.05 and a Log2FC value > 1 or < -1 were classified as being significantly differentially expressed. The analysis of RNA sequencing data included a comparison of the groups on a HSD and a control diet, as well as between the HSD with AITC and the HSD only, separately for males and females. The analysis did not include a comparison between control diet and HSD containing AITC. The illustrations were created using R (R Core Team, 2025). The R packages ‘ggplot2’ (27), “ggsignif” (28) and ‘ggbreak’ (29) were applied to create bar charts. The packages ‘pheatmap’ (30) and ‘RColorBrewer’ (31) were used to generate heat maps. The package ‘openxlsx’ (32) was employed to import the data.

For survival analysis the Kaplan–Meier approach and a log-rank test were applied to test for significant differences. Median survival times were calculated and tested for significant differences by performing a Mann–Whitney test. Significance was accepted at p < 0.05. Statistical analyses of qPCR were performed using GraphPad Prism software (v10.1.2, GraphPad Software, San Diego, CA, USA). Mean values were compared using a nonparametric test (Kruskal-Wallis), followed by pairwise comparison using Dunn’s multiple comparisons test. Significance was accepted at p < 0.05.

### 2.9 Data analysis for RNA sequencing

The information on the dme04624 signaling pathway from the KEGG database (33) accessed on May 22, 2025 was used as the data basis for the genes associated with the Toll and/or Imd signaling pathway. The genes contained in the KEGG database were augmented by further AMPs, which were also discovered to belong to the Toll and/or Imd signaling pathways, as reported in (20).

## 3 Results

### 3.1 Effect of high sucrose diet and allyl-isothiocyanate on feed intake

**Figure 2:**
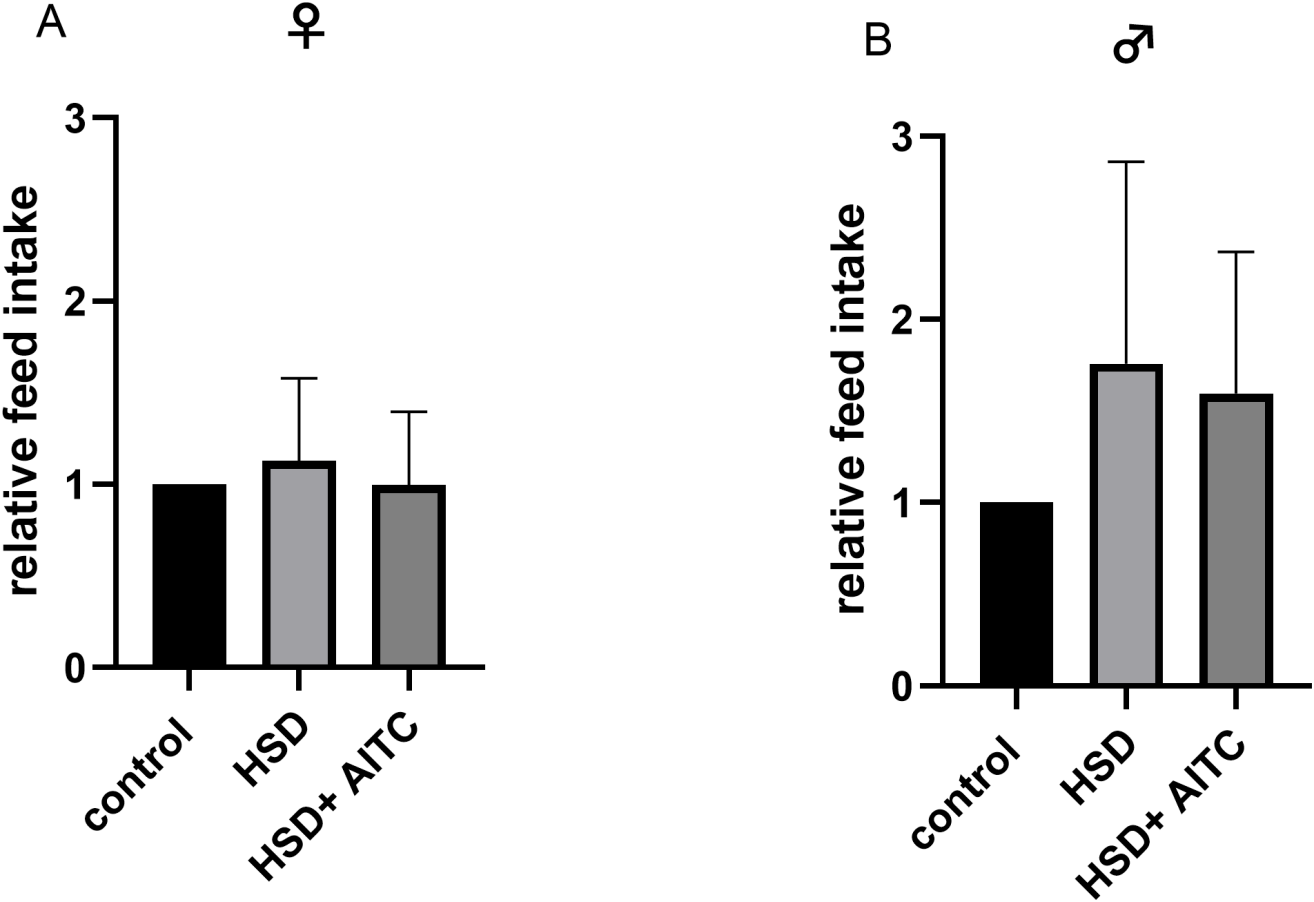
Relative feed intake of female **(A)** and male **(B)** *D. melanogaster* fed control diet (SY), high sucrose diet (HSD) or HSD containing 0.25 mM AITC (HSD+AITC) for 10 days. Significant differences between groups were tested by applying One-way ANOVA followed by a Tukey *post hoc* test. Three independent experiments (n=3), containing three vials, consisting of 20 flies each. Bars show the mean + SD.

To analyze the feed intake, flies were either fed a control diet, a HSD or a HSD containing 0.25 mM AITC for 10 days. As depicted in **Figure 2**, neither HSD, nor AITC significantly affected feed intake compared to the control diet. This was true for both, female and male flies (in female flies control vs. HSD p=0.8651, control vs. HSD + 0.25 mM AITC p=0.8183; in male flies control vs. HSD p=0.4311 and control vs. p=0.5721).

### 3.2 Effect of oral bacterial infections on the lifespan of *Drosophila melanogaster*

**Figure 3:**
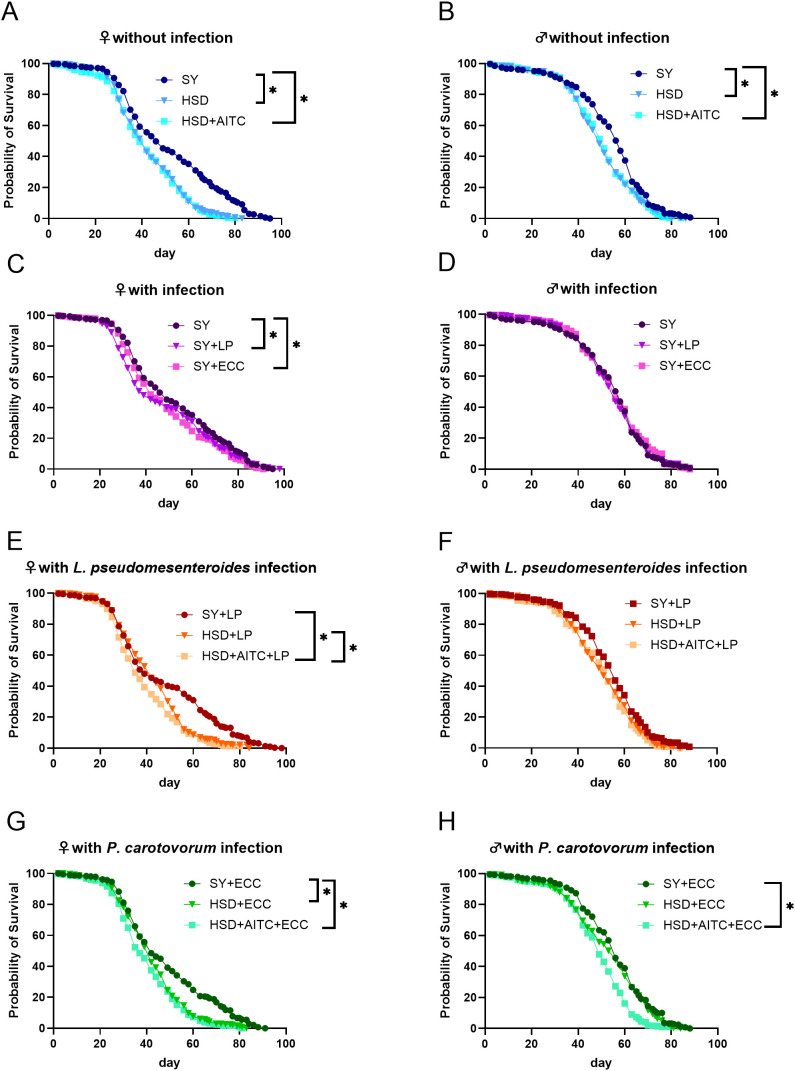
Survival curves of female **(A, C, E, G)** and male **(B, D, F, H)** *D. melanogaster* initially exposed to a control diet, high sucrose diet (HSD) or a HSD containing 0.25 mM AITC for 10 days. After 18 h of treatment either with sucrose solution or infection with LP, *L. pseudomesenteroides* (LP) or *P. carotovorum* subsp. *carotovorum* (ECC), flies were subsequently transferred to control diet (SY) or high sucrose diet. Results show the five independent experiments (n=5). Each experiment consisted of three vials per treatment group, containing 25 flies each. Significant differences between treatments were tested by the Kaplan–Meier approach and a log-rank test. Significance was accepted at *p < 0.05. **p < 0.01, ***p < 0.001, ****p < 0.0001.

Lifespan analysis revealed, that in female and male flies a HSD with and without AITC significantly shortened the lifespan (**Figures 3A, B**). Female flies which were infected with LP or ECC showed a significantly reduced life span, whereas in male flies no impact on survival was observed (**Figures 3C, D**). Female flies fed with HSD containing AITC had a significantly shorter lifespan in case of infection, compared to flies fed with control diet or high sucrose diet without AITC (**Figures 3E, F**). In LP infected male flies, no significant differences between lifespans were detected (**Figure 3F**). ECC infection resulted in a shorter lifespan in female flies fed a HSD or HSD containing AITC compared to female flies on a control diet (**Figure 3G**). In male flies, HSD+AITC significantly shortened the lifespan following infection with ECC compared to animals on a control diet and a HSD without AITC (**Figure 3H**). Comparison of median lifespan revealed no significant differences (**Supplementary Figure 7**).

### 3.3 Effects of high sucrose diet and allyl-isothiocyanate on gene expression of the Toll and Imd pathway

The RNA seq analysis revealed 50 genes belonging to the Toll pathway and 55 genes belonging to the Imd pathway, respectively. The encoding genes *AttA*, *Attacin B* (*AttB*)*, Cecropin A1* (*CecA1*)*, Cecropin A2 (CecA2), Cecropin B (CecB), Cecropin C (CecC), Peptidoglycan recognition protein SA* (*PGRP-SA*), *Peptidoglycan recognition protein SC1A* (*PGRP-SC1a*), *Peptidoglycan recognition protein SC1b* (*PGRP-SC1b*)*, Peptidoglycan recognition protein SC2* (*PGRP-SC2*) could be assigned to both signaling pathways.

**Figure 4:**
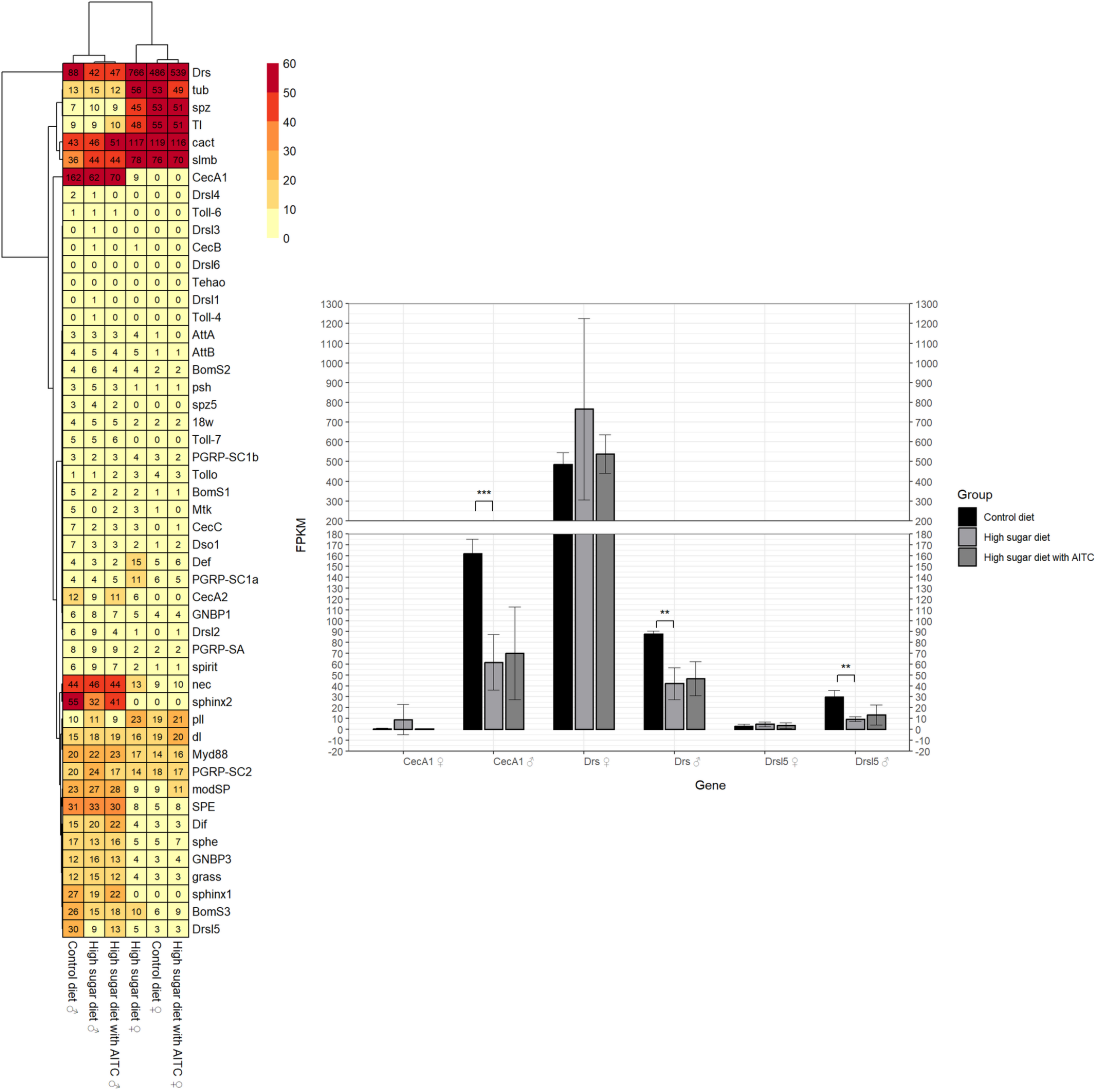
Heat map with FPKM values (mean of three replicates) for all genes of the Toll signaling pathway (left) and bar chart with mean FPKM values (± SD) of the significantly different genes for at least one group comparison in one sex (right); AITC, allyl-isothiocyanate; FPKM, Paired Fragments Per Kilobase of transcript per Million mapped reads; SD, standard deviation; Legend: ** adjusted p-value < 0.01; *** adjusted p-value < 0.001.

The FPKM (fragments per kilobase of transcript per million mapped reads) values for all genes of the Toll signaling pathway are shown for the three treatment groups and separated by sex in **Figure 4** (left). Statistically significant differences between HSD and control diet occurred exclusively in males, but not in females. Under HSD a downregulation of *CecA1*, *Drs* and *Drosomycin like 5* (*Drsl5*) was observed, with a Log2FoldChange of -1.43, -1.09 and -1.70, respectively (see **Figure 4**, right). AITC, when added to the HSD, exhibited no significant effect on the expression of Toll signaling pathway genes in either male or female subjects. An additional biological representation illustrating the significantly altered genes for the Toll signaling pathway can be found in **Supplementary Figure 5**.

**Figure 5:**
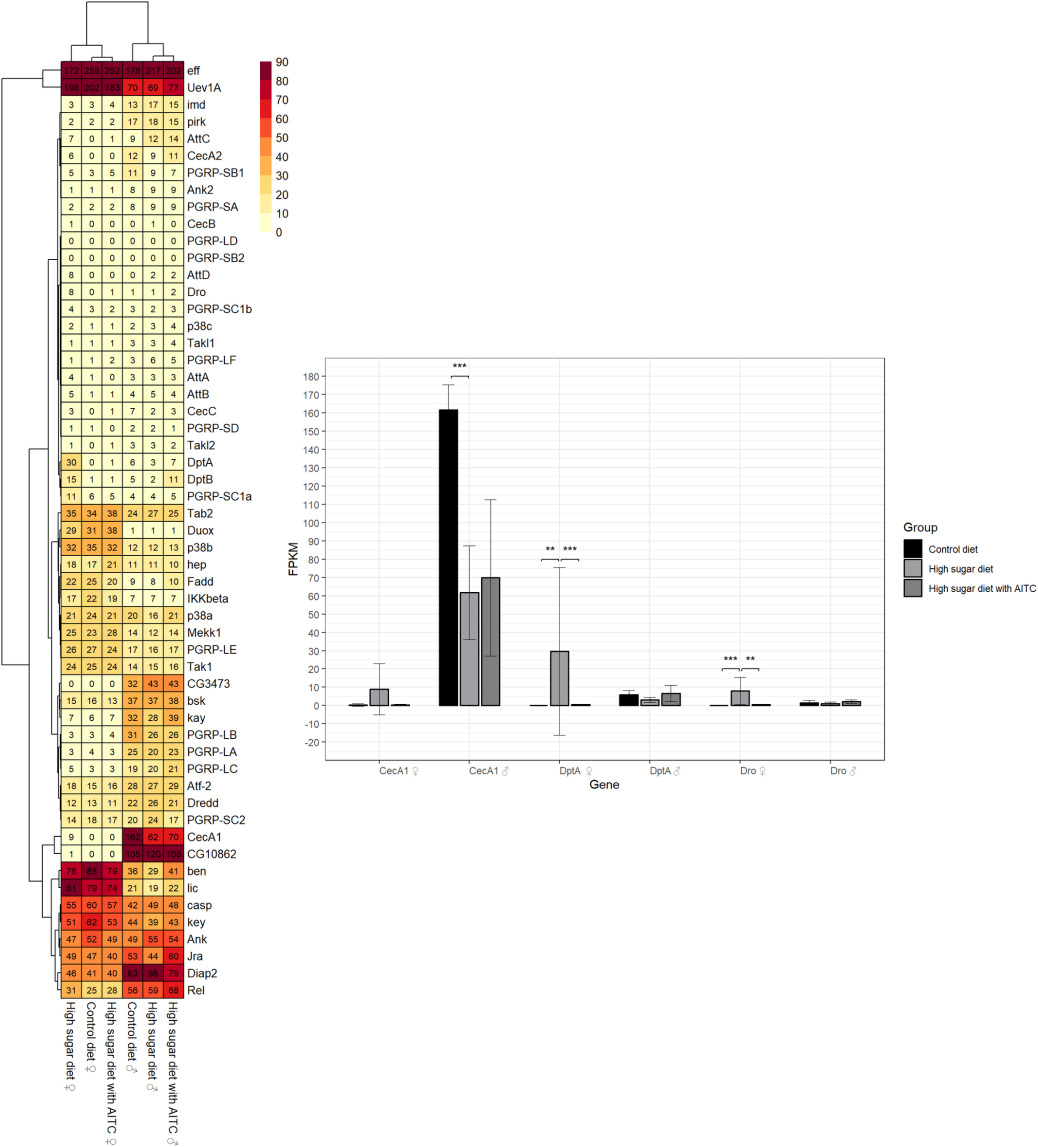
Heat map with FPKM values (mean of three replicates) for all genes of the Imd signaling pathway (left) and bar chart with mean FPKM values (± SD) of the significantly different genes for at least one group comparison in one sex (right); AITC, allyl-isothiocyanate; FPKM, Paired Fragments Per Kilobase of transcript per Million mapped reads; SD, standard deviation; ** adjusted p-value < 0.01; *** adjusted p-value < 0.001.

The heat map in **Figure 5** (left) indicates the FPKM values for all genes within the Imd signaling pathway. The bar chart on the right (**Figure 5**) illustrates the genes that have been identified as having statistically significant differences. In comparison with the control diets, the HSD resulted in the downregulation of *CecA1* (Log2FoldChange: -1.43) in males, a gene that is associated with both, Toll and Imd signaling pathway. Conversely, the female flies under HSD exhibited an upregulation of *DptA* and *Dro*, with a Log2FoldChange of 12.35 and 7.02, respectively. While the AITC added to the HSD had no effect on males, it led to a downregulation of *DptA* and *Drosocin* (*Dro*) in females, with a Log2FoldChange of -5.64 and -3.83, respectively. An additional biological representation illustrating the significantly altered genes for the Imd signaling pathway can be found in **Supplementary Figure 4**.

In summary, the RNA-seq data did not reveal coordinated regulation of genes belonging to the Toll or Imd signaling pathways. As shown in **Figures 4**, **5**, individual pathway components displayed heterogeneous fold-change patterns, with some genes being upregulated while others were downregulated, and with differences between males and females.

### 3.4 Combined effects of a high sucrose diet, allyl-isothiocyanate, and oral bacterial infection on AMP mRNA expression levels

**Figure 6:**
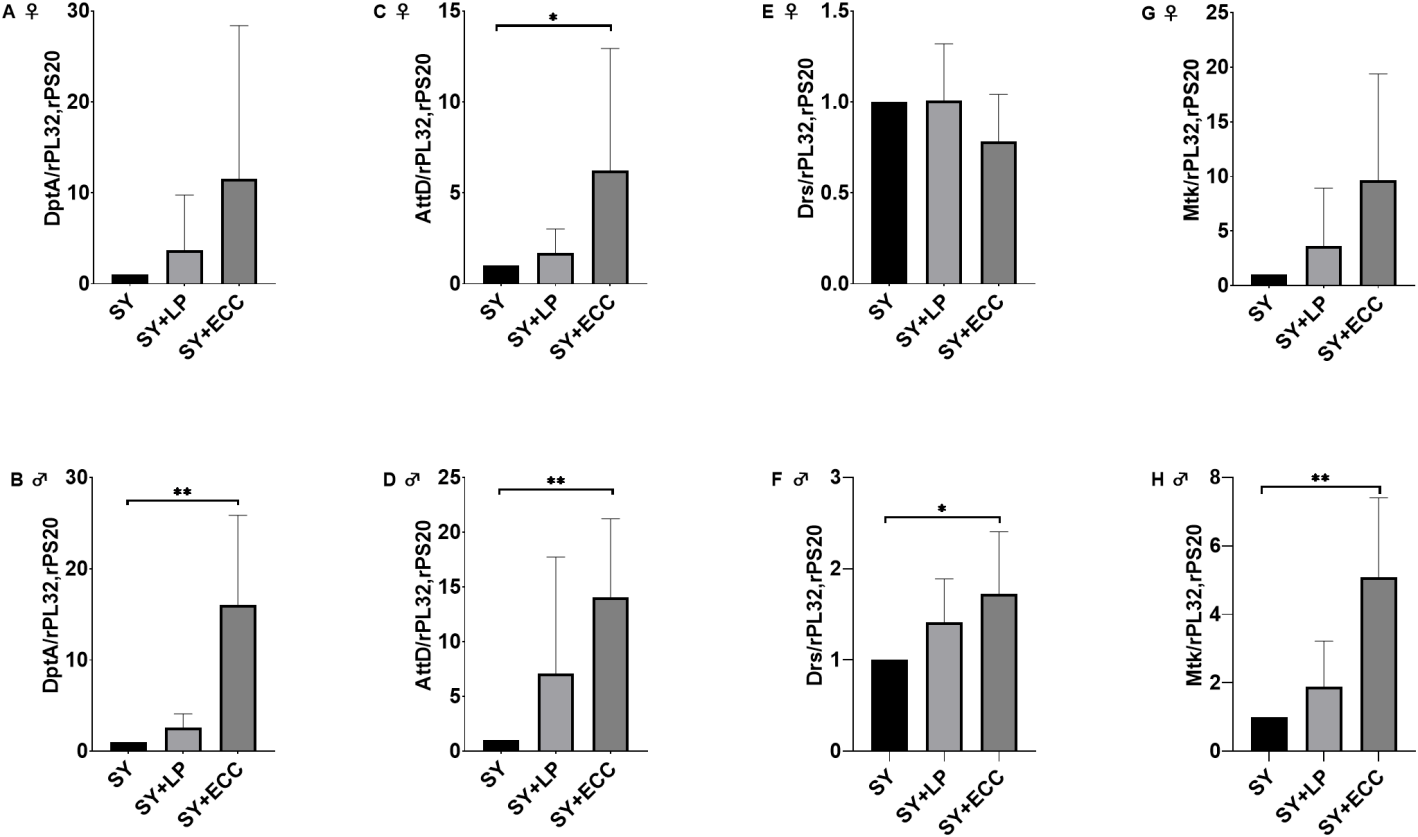
Relative mRNA expression levels of *Diptericin* (*DptA*) **(A, B)**; *Attacin D*, (*AttD*) **(C, D)**; *Drosomycin* (*DrsI*) **(E, F)**; and *Metchnikowin* (*Mtk*) **(G, H)** in female **(A, C, E, G)** and male (**B**, **D**, **F**, **H**) *D. melanogaster* exposed to a 100-mM sucrose solution without or with *P. carotovorum* subsp. *carotovorum* (ECC) or LP, *L. pseudomesenteroides* for 18 h after 10 days feeding on a control diet (SY). mRNA levels were determined in fly samples from five independent experiments containing 10 flies each, n=5. Bars show the mean + SD. Mean values were compared using a nonparametric test (Kruskal-Wallis), followed by pairwise comparison using Dunn’s multiple comparisons test. Significance was accepted at p < 0.05. * p<0.05, ** p< 0.005.

To investigate the impact of HSD and AITC on the expression of the AMPs *DptA*, *Mtk*, *AttD* and *Drs*, qPCR analysis was performed. Under non-infected conditions, neither HSD nor AITC affected the expression of these genes (**Supplementary Figure 1**). In LP-infected male and female flies on control diet, the expression levels of *DptA* were not affected (**Figures 6A, B**). In contrast, infection with ECC resulted in a significantly higher *DptA* expression in males. The same effect could be observed for Drs and Mtk (**Figures 6E-H**). Concerning *AttD*, infection with LP had no effect on the expression levels, whereas infection with ECC increased the expression levels in both, male and female flies (**Figures 6C, D**). Taken together, in flies fed with control diet an LP-induced infection had no impact on AMP expression, while an ECC infection resulted in significantly increased expression levels of *DptA*, *Mtk*, *AttD* and *Drs*, in a sex specific manner.

**Figure 7:**
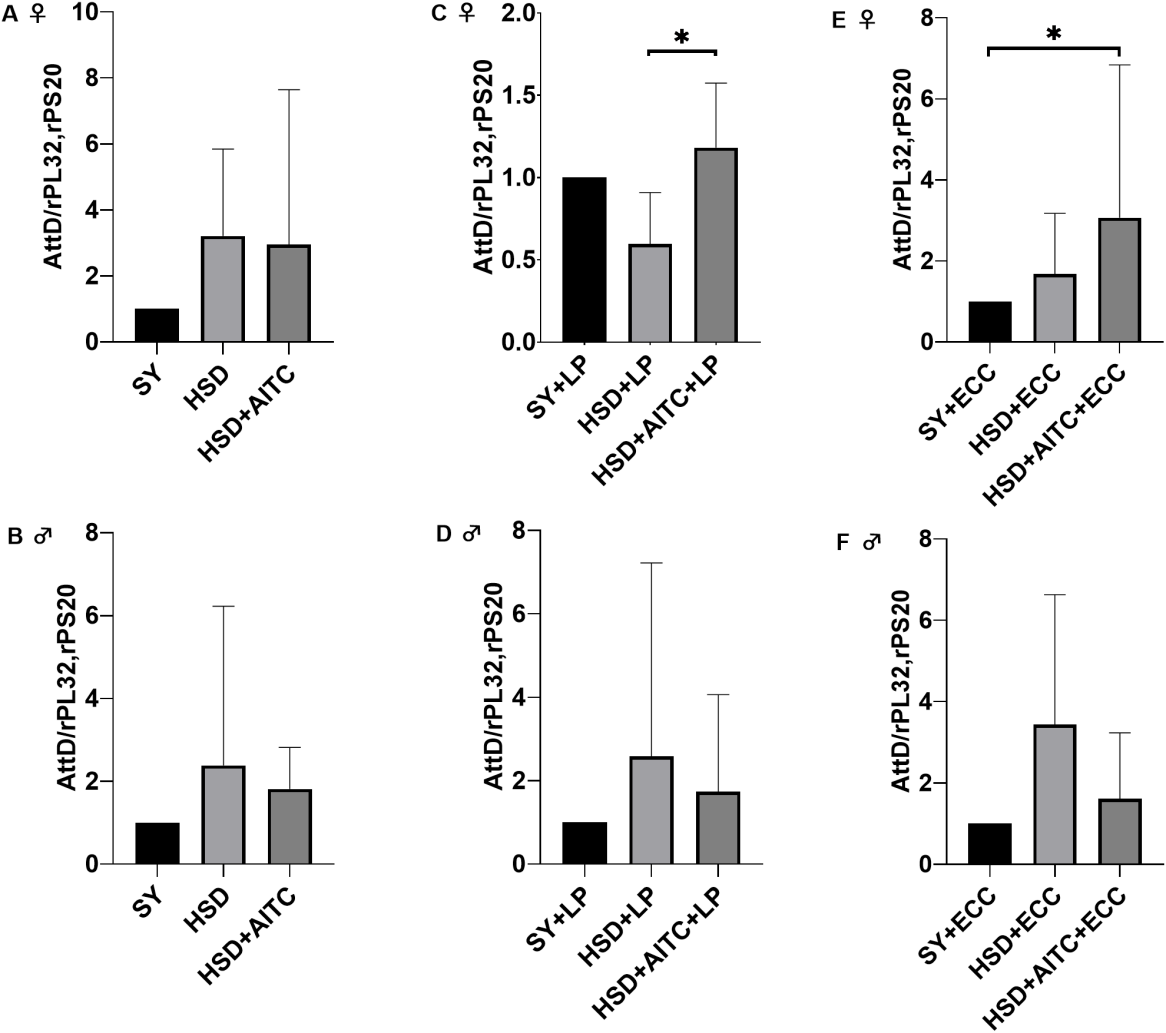
Relative mRNA expression levels of *Attacin D* (*AttD*) in female **(A, C, E)**, and male **(B, D, F)** *D. melanogaster* exposed to a 100-mM sucrose solution without or with *P. carotovorum* subsp. *carotovorum* (ECC) or *L. pseudomesenteroides* (LP) for 18 h after 10 days feeding on either a control diet (SY) or on a high sucrose diet (HSD) or a HSD supplemented with AITC (0.25 mM). mRNA levels were determined in fly samples from five independent experiments containing 10 flies each, n=5. Bars show the mean + SD. Mean values were compared using a nonparametric test (Kruskal-Wallis), followed by pairwise comparison using Dunn’s multiple comparisons test. Significance was accepted at p < 0.05. * p<0.05.

Under non-infected conditions, no differences in *AttD* expression levels were detected in HSD as well as in HSD+AITC fed female and male flies (**Figures 7A, B**). Under LP-infection, female flies fed a HSD supplemented with AITC exhibited significantly increased *AttD* expression levels, compared to flies fed HSD only (**Figure 7C**). In LP-infected male flies, no effect was observed (**Figure 7D**). In ECC-infected female flies on HSD+AITC, a significantly higher *AttD* expression was detected compared to flies receiving the control diet (**Figure 7E**). In ECC-infected male flies, no effect on *AttD* was present (**Figure 7F**).

The expression levels of *Mtk*, *DptA* and *Drs* were not changed in ECC- and LP- infected female and male flies following either HSD or HSD+AITC feeding (**Supplementary Figures 2**, **3**, **4**).

## 4 Discussion

AMPs have increasingly been considered promising candidates for alternative therapeutic strategies against bacterial infections (1). Western style diet, characterized, among other things, by an increased sugar consumption (34), and obesity have been discussed to induce a dysregulation of AMP production (12–14). As a potential candidate to affect bacterial infections and AMP production ITCs have been discussed (10). It has previously been shown that a supplementation of AITC to a standard laboratory fly diet mediates sex-specific effects on the survival of flies without altering the expression levels of several AMPs (23). Based on this, we hypothesized that AITC modulates AMP expression and survival outcomes in the context of a HSD in *D. melanogaster*. To address this, we added 0.25 mM AITC to HSD, as this concentration represents the highest tolerated and consumed dose, while higher concentrations lead to reduced feeding and analyzed the effect on AMP mRNA expression levels and survival under infectious and non-infectious conditions in *D. melanogaster* using qPCR, RNA sequencing, and lifespan assays. A gustatory assay confirmed that neither HSD nor AITC affected feed intake in these flies, indicating that the observed effects cannot be attributed to differences in ingestion. Instead, flies on HSD potentially consumed more calories, which likely contributed to the reduced lifespan observed in both sexes, being consistent with previous reports describing the detrimental effects of HSD on longevity (35). The supplementation of HSD with AITC did, however, not rescue the reduced lifespan caused by HSD. Moreover, HSD affected the fly survival rates following bacterial infection in a sex- and pathogen-specific manner. While HSD had no impact on post-infection survival rates in males, it reduced survival in females following ECC infection but not LP infection. These findings are in contrast to previous studies using systemic infection fly models, in which high-sugar or high-glucose diets increased the bacterial load and mortality *in D. melanogaster* after infection with *Providencia rettgeri* or *Serratia marcescens* (36, 37). These discrepancies may be explained by differences in the infection routes. In contrast to systemic infection resulting from bacterial injection, oral infection triggers distinct local and systemic immune responses, as well as by pathogen-specific effects. The relatively modest changes in AMP expression observed in this study likely reflect a low-grade modulation of immune signaling rather than a full activation of the immune response, as typically seen during acute systemic infections. Importantly, our data further highlight sex-specific differences in diet–infection interactions, which remain underexplored, as many studies focus on only one sex (38). Although the reproductive output was not directly measured in the present study, the observed sex-specific mortality pattern may be compatible with a reproduction–immunity trade-off, a well-established concept in insect life-history theory (38–40).

With respect to AITC, we observed pronounced sex-specific effects on survival after infection. Following LP infection, AITC shortened the lifespan in females but had no effect in males, whereas after ECC infection, AITC significantly reduced survival in males but not in females. Despite its well-known antimicrobial activity *in vitro* (7), AITC did not improve fly survival following bacterial infections. Instead, whenever AITC showed a significant effect, it was associated with a shortened lifespan. This pattern is consistent with previous findings demonstrating sex-specific and detrimental effects of AITC during infections in *D. melanogaster* (7, 9, 23), underscoring that *in vitro* antimicrobial activity does not necessarily translate into improved host survival *in vivo*.

Since the induction of AMP expression represents a central readout of antibacterial immunity in *D. melanogaster*, we first investigated whether an oral bacterial infection triggered the expression of several AMPs in control fed flies. We observed a clear sexual dimorphism: in female flies, bacterial infection did not significantly alter the expression of *DptA*, *Drs* or *Mtk* (**Figure 6**) and resulted in a shortened lifespan (**Figure 3**). In contrast, male flies exhibited increased AMP expression levels after bacterial infection without a detectable cost of survival. One possible explanation is that infection-induced immune activation in males may contribute to a more effective control of bacterial proliferation, thereby preventing a decrease in lifespan, whereas the absence of a detectable systemic infection-induced AMP expression in females may reflect an impaired resistance. However, this hypothesis requires a direct validation, for example through measurements of bacterial loads over time or by the use of transgenic flies with impaired AMP responses. It should also be considered that oral infection primarily triggers local gut immune responses, which may not be fully captured by systemic AMP measurements.

Second, we investigated the effect of HSD and AITC on AMP expression. Neither HSD nor AITC altered basal AMP expression in the absence of infection (**Figures 7A, B**). Third, we examined the combination of infection with HSD and/or AITC. In males, no changes in AMP expression levels were detected following bacterial infection (**Figure 7**; **Supplementary Figures 2**, **3**, **4**), suggesting that the observed effects of HSD and AITC on survival are unlikely to be mediated by altered AMP transcription. Instead, diet and AITC may influence infection outcomes through alternative mechanisms, including enhanced pathogen growth under high-sugar conditions, metabolic alterations of the host, changes in epithelial barrier function, or a modulation of immune signaling dynamics. Indeed, Darby et al. (2024) (37) demonstrated that high-sugar diets are able to promote bacterial proliferation in *D. melanogaster*, leading to increased mortality which is independent of AMP transcript levels.

Interestingly, in female flies AITC increased *AttD* expression after infection (**Figure 7E**), coinciding with reduced survival. This suggests that induction of AMP expression under these conditions may represent a compensatory or insufficient immune response rather than an effective protection. RNA sequencing further revealed diet- and AITC-dependent regulation of several AMPs, while no transcriptional changes were detected in core components of the Toll or Imd pathways (**Figures 4**, **5**). This is consistent with the fact that activation of immune signaling pathways is predominantly regulated at the post-translational level, including phosphorylation, proteolytic processing, and nuclear translocation of transcription factors, such as proteolytic activation of Relish in the Imd pathway (41). In addition, epigenetic mechanisms and post-transcriptional regulation, including altered mRNA stability, microRNA-mediated control, or alternative polyadenylation, may contribute to a context-dependent modulation of AMP expression without changes in upstream pathway gene expression levels (42, 43).

Although a general toxic effect of AITC cannot be fully excluded, the context-dependent nature of the observed effects, which became apparent primarily under infection conditions, argues against a purely unspecific toxicity. One limitation of the present study is that immune responses as AMP expression levels were only assessed at the transcriptional level. Future studies would benefit from a quantification of AMP expression at the protein level, as dietary effects on the immune defense may only become apparent post-transcriptionally. Indeed, Darby et al. (37) showed that a dietary modulation during *P. rettgeri* infection affected Drosocin protein levels without altering the transcript abundance. Due to moderate alterations in AMP expression levels, it seems to be unclear, whether the oral infection induced a biological relevant activation of Toll and IMD signaling. Therefore, using a stronger activator of these pathways would be helpful to resolve the impact of dietary AITC. Moreover, in the present study only a limited number of AMPs was analyzed by qPCR; expanding this panel would provide a more comprehensive view of the humoral immune regulation. Additionally, gene expression analyses were restricted to a single time point (18 h post infection). As immune responses are highly dynamic, additional time-course analysis would be required to fully resolve the temporal regulation of AMP expression (44). Although our data identify specific antimicrobial peptides as potential mediators of the observed AITC effects, causality cannot be inferred from correlative expression analyses alone. Future studies should include genetically modified flies with targeted AMP knockouts or overexpression constructs, to determine whether these peptides directly drive the survival phenotypes observed in this study. Although the use of *D. melanogaster* as a model organism is limited, as insect physiology and immune responses differ from those of mammals and therefore limit direct translation to human biology. However, key components of innate immune signaling as well as fundamental interactions between nutrition and immune function are evolutionarily conserved, making *D. melanogaster* a powerful model for investigating basic mechanisms of diet–immune interactions. Together, our findings indicate that AITC and dietary sugar modulate bacterial infection outcomes in a sex-specific manner through mechanisms beyond simple transcriptional regulation of canonical immune effectors.

## Data Availability

The datasets presented in this study can be found in online repositories. The names of the repository/repositories and accession number(s) can be found in the article/**Supplementary Material**.
