# Supplementary material for: Impact of allyl-isothiocyanate and high sucrose diet on antimicrobial peptide expression and survival in Drosophila melanogaster
Source: Front Immunol. 2026 Apr 10;17:1804605. doi: 10.3389/fimmu.2026.1804605 (PMC13106523; doi:10.3389/fimmu.2026.1804605)
Supplement: Supplementary file 1 [file DataSheet1.pdf]

Supplementary material

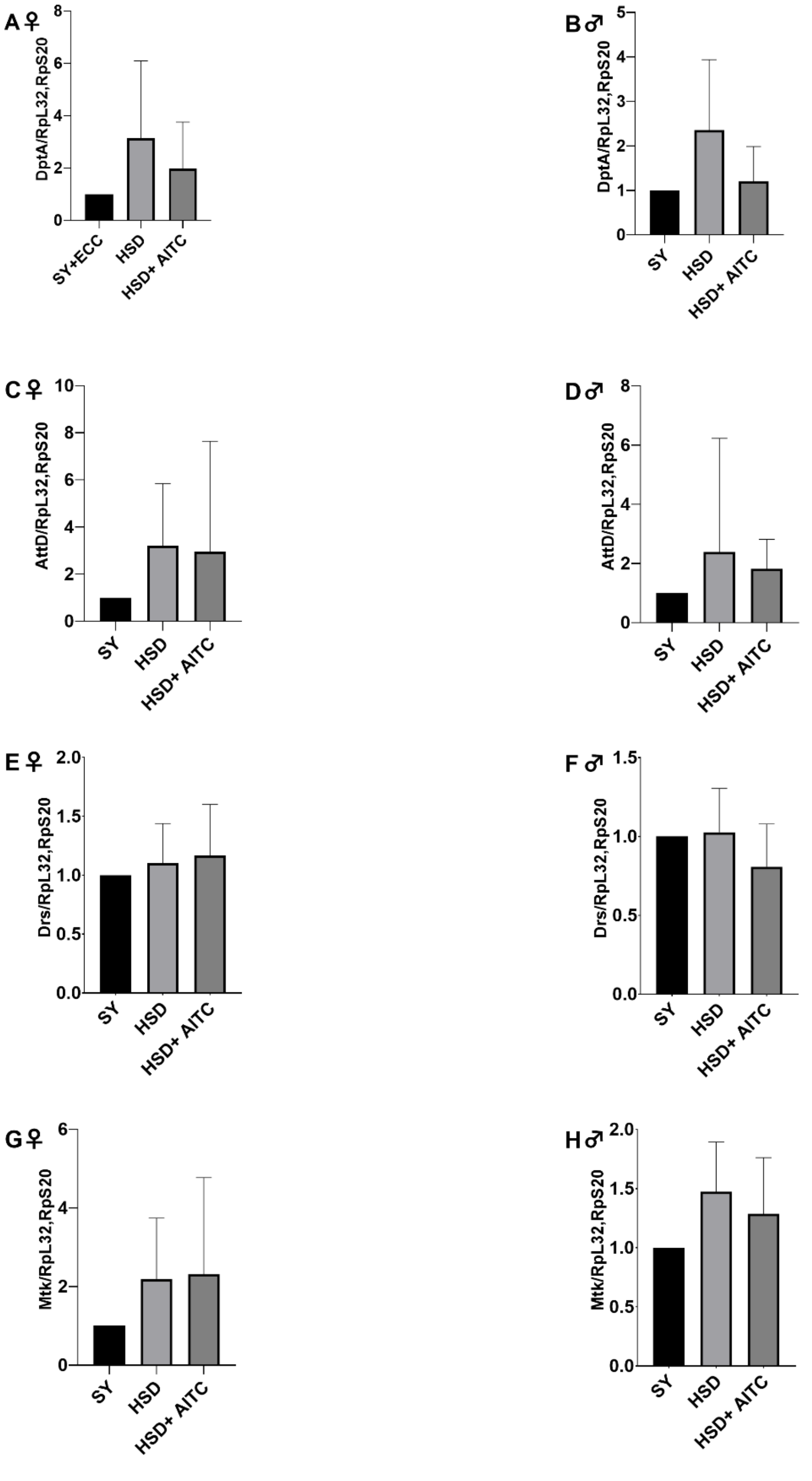

Supplementary figure 1: Relative mRNA expression levels of *Diptericin* (*DptA*, **A**, **B**), *Attacin D* (*AttD*, **C**, **D**), *Drosomycin* (*Drs*, **E**, **F**) and *Metchnikowin* (*Mtk*, G, H) in female **(A, C, E, G))** and male **(B, D, F, H)** *D. melanogaster* after 10 days feeding on a control diet (SY), highs sucrose diet (HSD) or high sucrose diet containing 0.25 mM AITC (HSD+AITC). mRNA levels were determined in fly samples from five independent experiments containing 10 flies each. Bars show the mean + SD. Mean values were compared using a nonparametric test (Kruskal-Wallis), followed by pairwise comparison using Dunn's multiple comparisons test. Significance was accepted at $p < 0.05$.

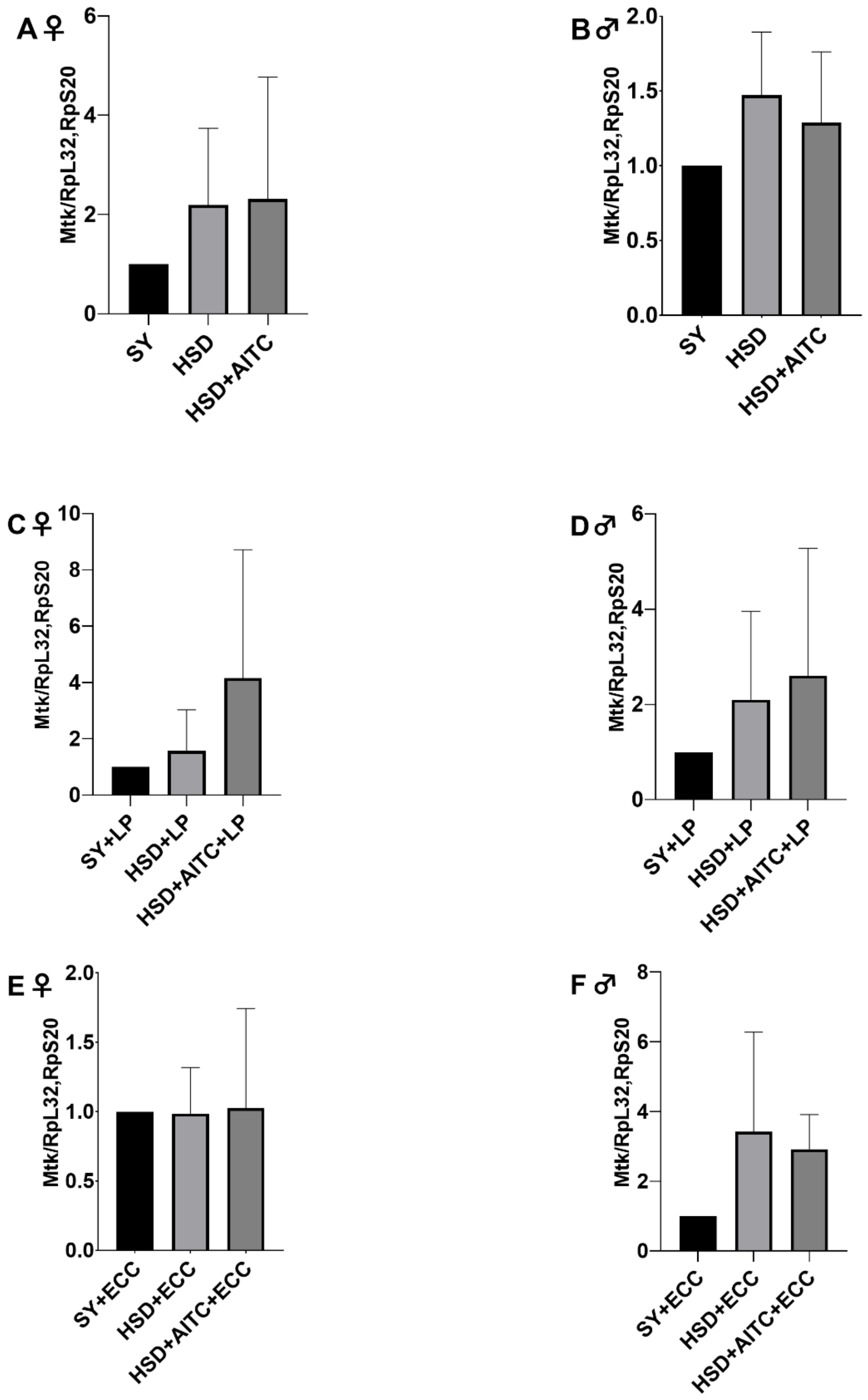

Supplementary figure 2: Relative mRNA expression levels of *Metchnikowin* (*Mtk*) in female **(A, C, E,)** and male **(B, D, F)** *D. melanogaster* exposed to a 100-mM sucrose solution with *P. carotovorum* subsp. *carotovorum* (ECC) or *L. pseudomesenteroides* (LP) for 18 h after 10 days feeding on either a control diet or an high sucrose diet or an high sucrose diet supplemented with AITC (0.25 mM). mRNA levels were determined in fly samples from five independent experiments containing 10 flies each. Bars show the mean + SD. Mean values were compared using a nonparametric test (Kruskal-Wallis), followed by pairwise comparison using Dunn's multiple comparisons test. Significance was accepted at $p < 0.05$.

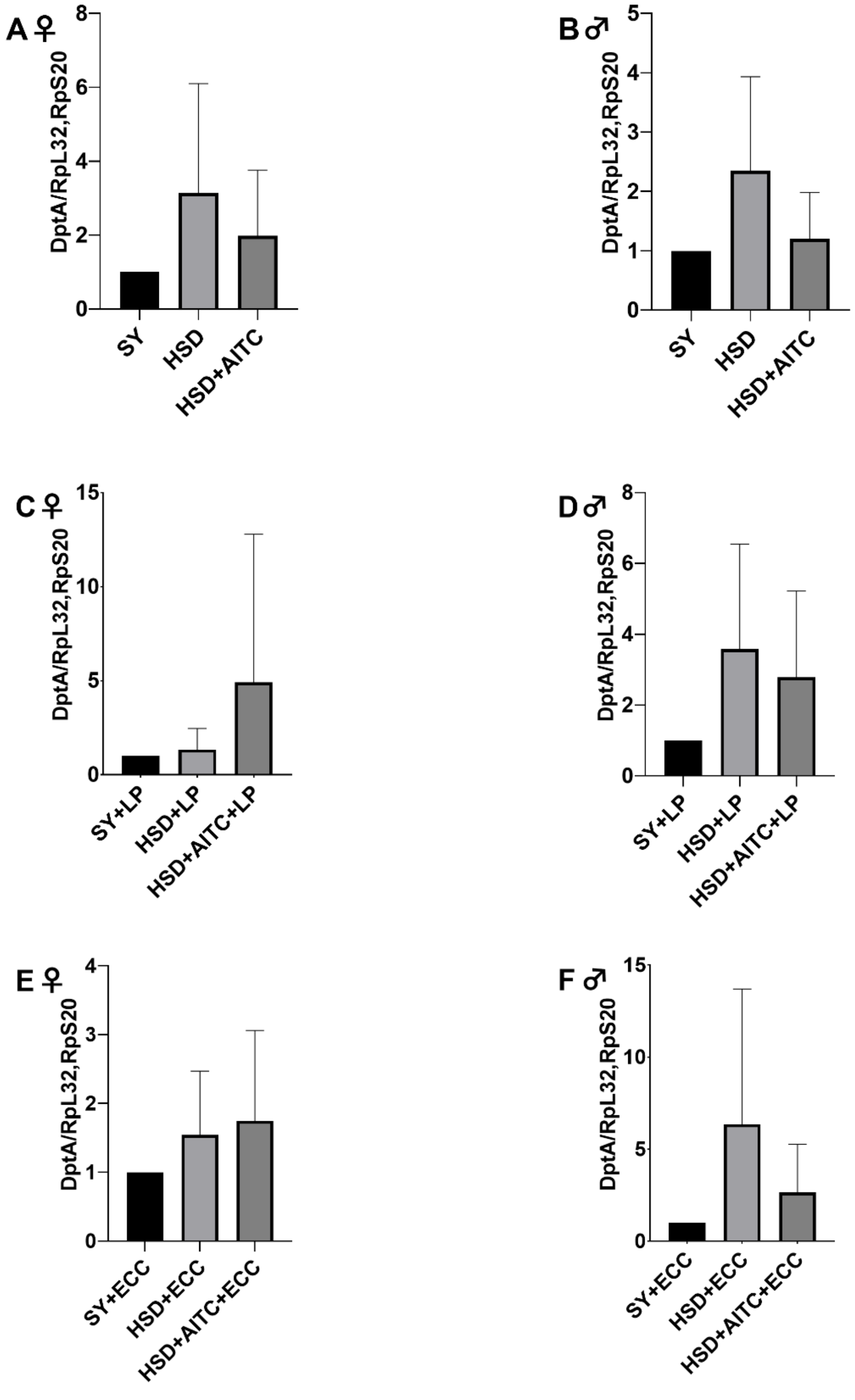

Supplementary figure 3: Relative mRNA expression levels of *Diptericin A* (*DptA*) in female **(A, C, E,)** and male **(B, D, F)** *D. melanogaster* exposed to a 100-mM sucrose solution with *P. carotovorum* subsp. *carotovorum* (ECC) or *L. pseudomesenteroides* (LP) for 18 h after 10 days feeding on either a control diet or an high sucrose diet or an high sucrose diet supplemented with AITC (0.25 mM). mRNA levels were determined in fly samples from five independent experiments containing 10 flies each. Bars show the mean + SD. Mean values were compared using a nonparametric test (Kruskal-Wallis), followed by pairwise comparison using Dunn's multiple comparisons test. Significance was accepted at $p < 0.05$.

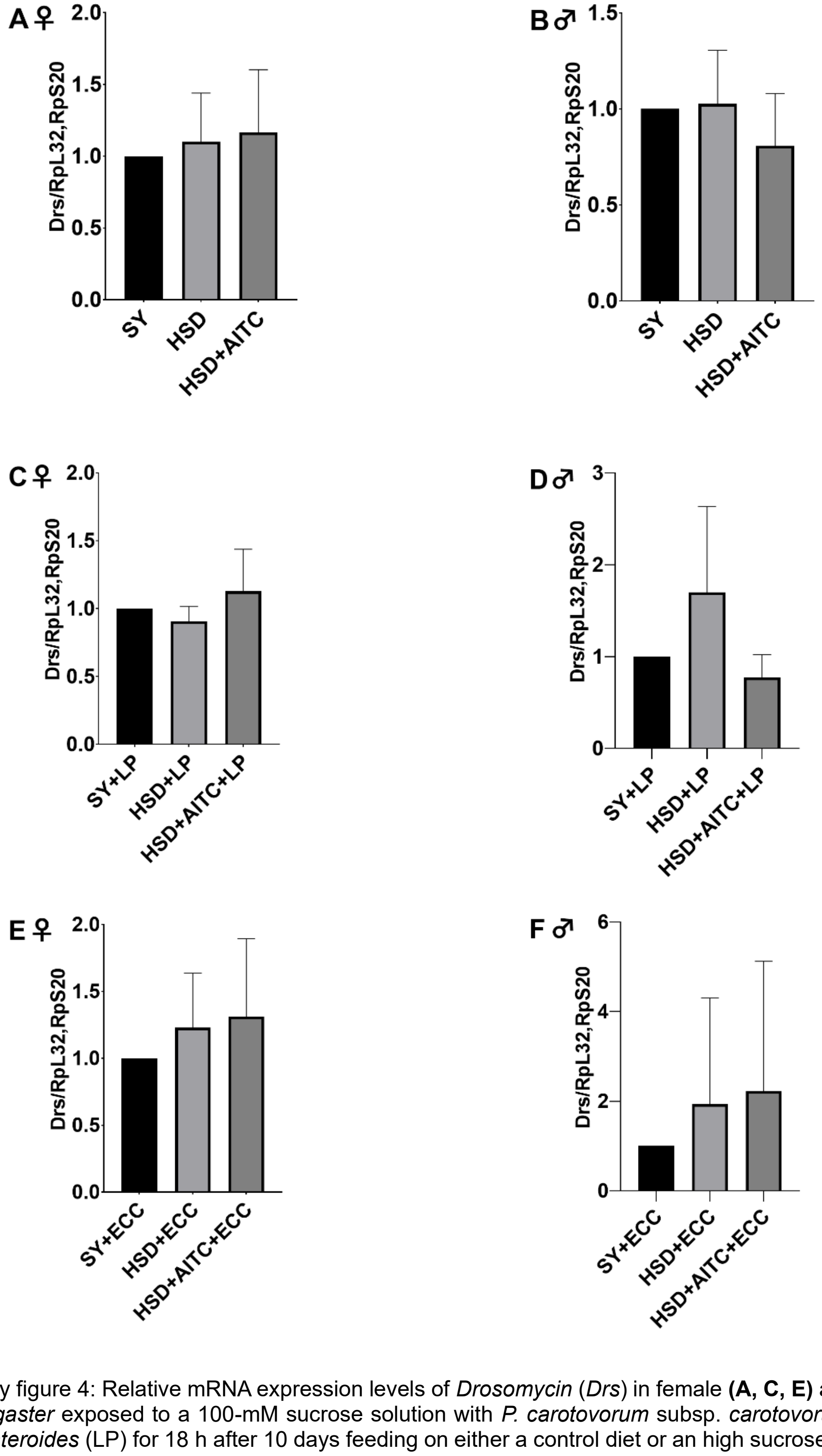

Supplementary figure 4: Relative mRNA expression levels of *Drosomycin* (*Drs*) in female **(A, C, E)** and male **(B, D, F)** *D. melanogaster* exposed to a 100-mM sucrose solution with *P. carotovorum* subsp. *carotovorum* (ECC) or *L. pseudomesenteroides* (LP) for 18 h after 10 days feeding on either a control diet or an high sucrose diet or an high sucrose diet supplemented with AITC (0.25 mM). mRNA levels were determined in fly samples from five independent experiments containing 10 flies each. Bars show the mean + SD. Mean values were compared using a nonparametric test (Kruskal-Wallis), followed by pairwise comparison using Dunn's multiple comparisons test. Significance was accepted at $p < 0.05$.

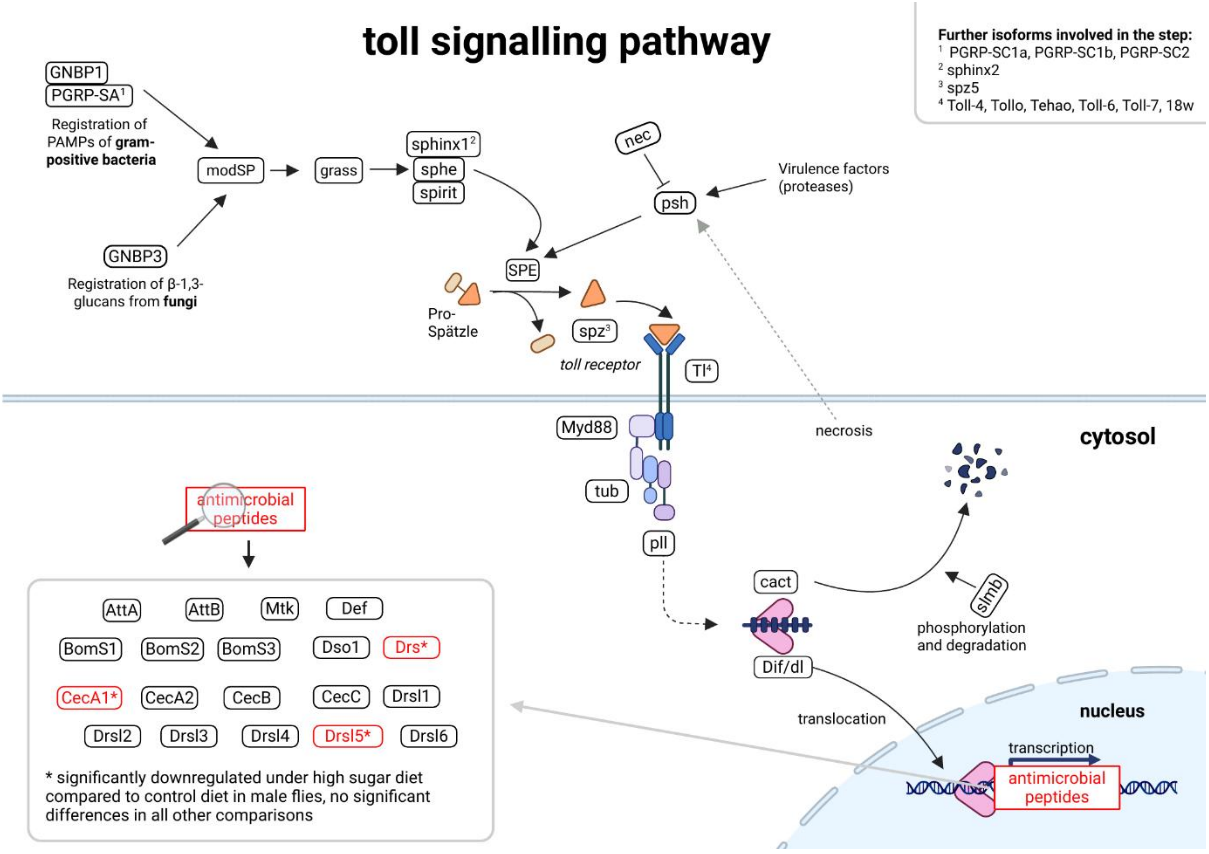

Supplementary figure 5: Key steps in the Toll signaling pathway (created with BioRender based on the BioRender template ‘Drosophila Toll pathway’ by Silverman und Ona; underlying information for the figure design also from Valanne et al. 2022 and Yu et al. 2022; **Abbreviations:** PAMPs, pathogen-associated molecular patterns. Created in BioRender. Dähn, S. (2026) https://BioRender.com/6zx1y8d

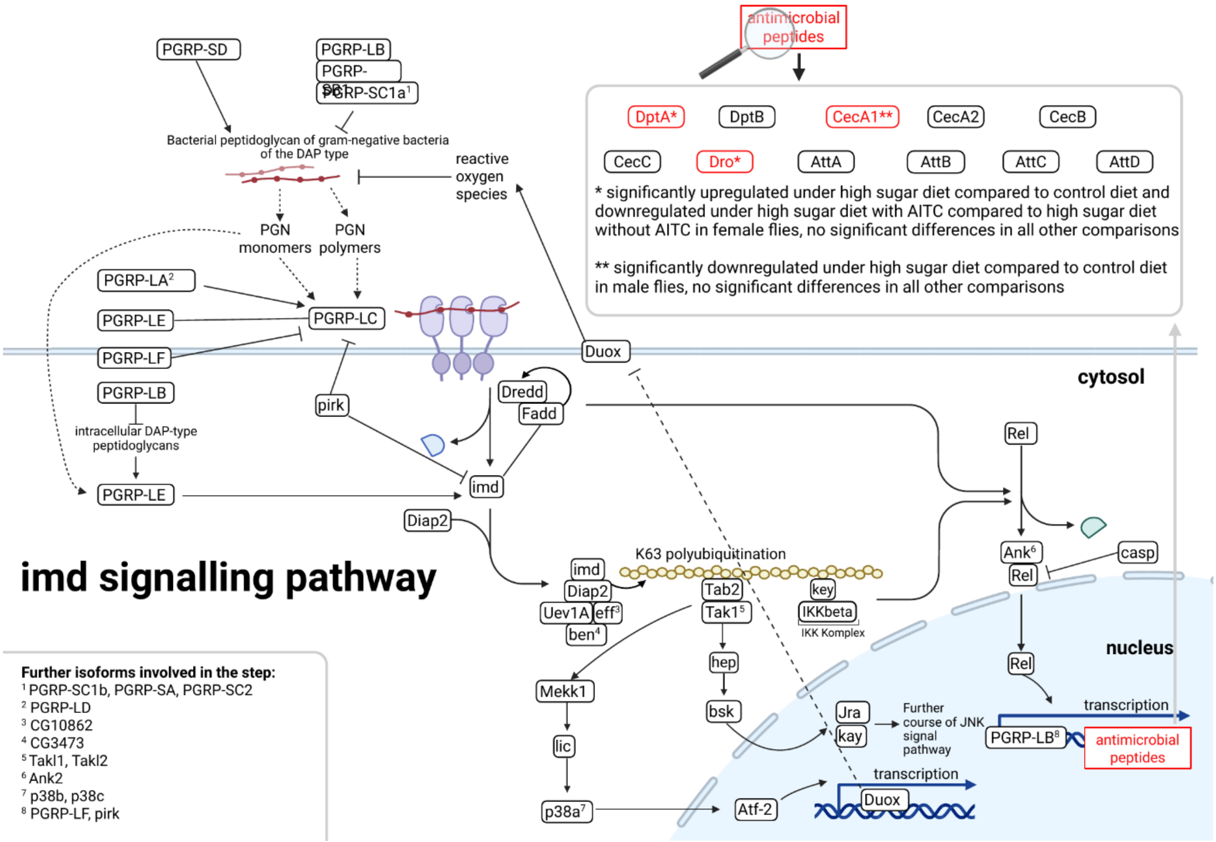

Supplementary figure 6: Key steps in the IMD signaling pathway (created with BioRender based on the BioRender template 'IMD Signaling Pathway' by Silverman und Kim; underlying information also from Yu et al. 2022; **Abbreviations:** DAP, diaminopimelic acid; PGN, peptidoglycan. Created in BioRender. Dähn, S. (2026) https://BioRender.com/v3386n6

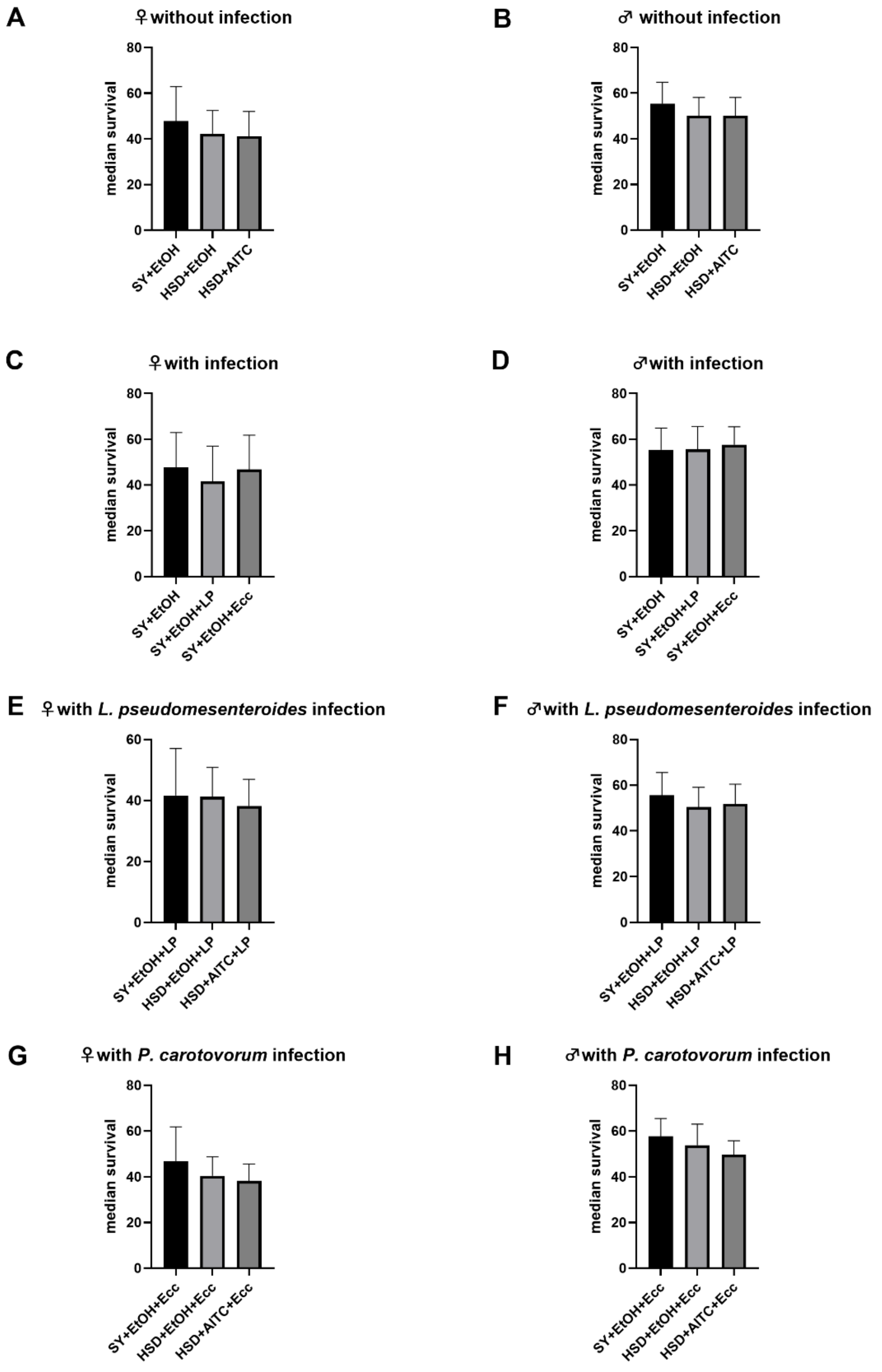

Supplementary figure 7: Median survival of female **(A, C, E)** and male **(B, D, F)** *D. melanogaster*, fed either control diet or diet supplemented with 0.25 mM AITC. A and B: without infection, C and D: after 18 h of oral infection with *P. carotovorum* subsp. *carotovorum* (ECC) or *L. pseudomesenteroides* (LP). E and F: after 18 h of oral infection with *L. pseudomesenteroides* (LP). G and H: after 18 h of oral infection with *P. carotovorum* subsp. *carotovorum* (ECC). The bars represent the mean (+SD) of five independent experiments, each containing 75 flies. Mean values were compared using ordinary one-way ANOVA, followed by Tukey´s multiple comparisons test. Significance was accepted at $p < 0.05$.

Literaturverzeichnis

Silverman, N.; Kim, S.: IMD Signaling Pathway. BioRender Template.

Silverman, N.; Ona, S.: Drosophila Toll Pathway. BioRender Template.

Valanne, Susanna; Vesala, Laura; Maasdorp, Matthew K.; Salminen, Tiina S.; Rämet, Mika (2022): The Drosophila Toll Pathway in Innate Immunity: from the Core Pathway toward Effector Functions. In: *Journal of immunology (Baltimore, Md. : 1950)* 209 (10), S. 1817–1825. DOI: 10.4049/jimmunol.2200476.

Yu, Shichao; Luo, Fangzhou; Xu, Yongyi; Zhang, Yan; Jin, Li Hua (2022): Drosophila Innate Immunity Involves Multiple Signaling Pathways and Coordinated Communication Between Different Tissues. In: *Frontiers in immunology* 13, S. 905370. DOI: 10.3389/fimmu.2022.905370.
